# A Preliminary Study of the Effectiveness of an Allostatic, Closed-Loop, Acoustic Stimulation Neurotechnology in the Treatment of Athletes with Persisting Post-concussion Symptoms

**DOI:** 10.1186/s40798-016-0063-y

**Published:** 2016-09-14

**Authors:** Charles H. Tegeler, Catherine L. Tegeler, Jared F. Cook, Sung W. Lee, Lee Gerdes, Hossam A. Shaltout, Christopher M. Miles, Sean L. Simpson

**Affiliations:** 1 Department of Neurology, Wake Forest School of Medicine, Medical Center Boulevard, Winston-Salem, NC 27157-1078 USA; 2 Brain State Technologies, LLC, Scottsdale, AZ USA; 3 Department of Obstetrics and Gynecology, Hypertension and Vascular Research Center, Wake Forest School of Medicine, Winston-Salem, NC USA; 4 Sports Medicine, Department of Family and Community Medicine, Wake Forest School of Medicine, Winston-Salem, NC USA; 5 Department of Biostatistical Sciences, Wake Forest School of Medicine, Winston-Salem, NC USA

**Keywords:** Sports concussion, Persisting post-concussion symptoms, Post-concussion syndrome, Allostasis, Neurotechnology, HIRREM, Baroreflex sensitivity, Heart rate variability, Reaction time, Return to play

## Abstract

**Background:**

Effective interventions are needed for individuals with persisting post-concussion symptoms. High-resolution, relational, resonance-based, electroencephalic mirroring (HIRREM®) is an allostatic, closed-loop, acoustic stimulation neurotechnology, designed to facilitate relaxation and self-optimization of neural oscillations.

**Methods:**

Fifteen athletes (seven females, mean age 18.1 years, SD 2.6) with persisting post-concussion symptoms received 18.7 (SD 6.0) HIRREM sessions over a mean of 29.6 (SD 23.2) days, including 11.3 (SD 4.6) in office days. Pre- and post-HIRREM measures included the Rivermead Post-Concussion Symptoms Questionnaire (RPQ, *n* = 12), the Insomnia Severity Index (ISI, *n* = 15), the Center for Epidemiologic Studies Depression Scale (CES-D, *n* = 10), short-term blood pressure and heart rate recordings for measures of autonomic cardiovascular regulation (*n* = 15), and reaction time by the drop-stick method (*n* = 7). All participants were asked about their physical activity level and sports participation status at their post-HIRREM data collection visit and 1 to 3 months afterward.

**Results:**

At the post-HIRREM visit, subjects reported improvements in all three inventories (RPQ mean change 19.7, SD 11.4, Wilcoxon *p* = 0.001; ISI mean change −4.1, SD 4.1, Wilcoxon *p* = 0.003; CES-D mean change −12.0, SD 10.0, Wilcoxon *p* = 0.004), including statistically significant reductions in 14 of the 16 individual items of the RPQ. There were also statistically significant improvements in baroreflex sensitivity, heart rate variability in the time domain (SDNN), and drop-stick reaction testing (baseline mean distance of 23.8 cm, SD 5.6, decreased to 19.8 cm, SD 4.6, Wilcoxon *p* = 0.016). Within 3 months of the post-HIRREM data collection, all 15 had returned to full exercise and workouts, and ten had returned to full participation in their athletic activity.

**Conclusions:**

The use of HIRREM by a series of athletes with persisting post-concussion symptoms was associated with a range of improvements including, for the majority, return to full participation in their sport. The findings do not appear to be consistent with constituents of the placebo effect. A larger controlled trial is warranted.

## Key Points

- Persisting post-concussion symptoms (PPCS) can be highly debilitating and refractory to therapy.
- Autonomic dysregulation and sleep disturbance are common with PPCS and may be related to trauma-induced hemispheric asymmetry as well as increased amplitudes in high frequency ranges of brain electrical activity.
- The use of a closed-loop allostatic, acoustic stimulation neurotechnology in a series of athletes with PPCS was associated with improvements in clinical symptoms and autonomic cardiovascular regulation, potentially mediated by shifting of brain electrical activity patterns in the direction of greater hemispheric symmetry and reduction of high-frequency amplitudes.
- Outcomes demonstrated in this study including a high rate of return to play do not appear to be consistent with anticipated natural history, or the placebo effect, and a larger controlled trial is merited.

## Background

Athletes are at risk for sports-related mild traumatic brain injury (mTBI) or concussion. A majority of concussed athletes recover quickly and may be eligible for return to athletic participation after they are asymptomatic [1]. It is well established, however, that a subset develops a post-concussion syndrome or PPCS [2]. PPCS disturbances include physical (headache, dizziness, fatigue, balance problems, sleep disturbance), cognitive (memory and attention problems), and emotional (depression, anxiety, irritability) challenges that persist beyond 7 to 10 days after the concussion, affecting approximately 10 to 15 % of concussed athletes [3]. A 2013 review of PPCS management highlighted that while many studies have now been published on acute concussion, much remains unknown about optimal care for those with prolonged symptoms [4].

Supporting improved autonomic nervous system (ANS) regulation may be a strategic way to aid return to physical activity for athletes with PPCS. The ANS has pervasive influences on cardiovascular, pulmonary, gastrointestinal, immune, cognitive, emotional, and behavioral systems [5], and ANS dysregulation is a likely major pathway for the range of symptoms in mTBI [6]. Studies have reported that mTBI is associated with lower heart rate variability (HRV) [7, 8], indicating a loss of dynamic flexibility in the capacity of the ANS to optimize or fine-tune cardiovascular regulation. A subset of individuals with TBI subsequently manifests paroxysmal sympathetic hyperactivity [9, 10] which presents with symptoms of tachycardia, hypertension, tachypnea, and diaphoresis. Cerebral auto-regulation, the capacity of the cerebral vasculature to maintain intra-cranial blood pressure within a narrow range, is compromised in up to 30 % of patients with mTBI, and it is likely to depend critically on autonomic mechanisms [11–13].

The aim of the present study was to evaluate the potential role of high-resolution, relational, resonance-based electroencephalic mirroring (HIRREM®; Brain State Technologies, Scottsdale, AZ), a noninvasive, closed-loop, acoustic stimulation neurotechnology [14], as a means to support symptom reduction, improved autonomic function, and return to play among athletes with PPCS. HIRREM is designed to support auto-calibration of neural oscillations by recording brain electrical activity at high spectral resolutions, applying algorithms to analyze oscillatory patterns, translating selected electrical frequencies into sonic frequencies, and returning audible tones of variable pitch and timing back to the user, in real time. In contrast to conventional homeostatic therapeutics, HIRREM is aligned with the *allostasis* paradigm of physiological regulation [15, 16]. Allostasis views disease not as the product of fundamental abnormality of given biological mechanisms but rather as the relative persistence or rigidity of system set points that renders systems (and the organism as a whole) less capable of dynamic adaptation. Health is a capacity for successful engagement with changing conditions of the natural environment, and the brain is the seat for orchestration of various system functions in concert.

In that its intended use is to support individually unique and adaptively changing set points for brain electrical activity, HIRREM is an allostatic modality for health recovery. Auto-calibration of neural oscillations may manifest as reduction of maladaptive forms of asymmetry between the right and left hemispheres [14], in multiple lobes including regions likely to play a role for management of the sympathetic and parasympathetic divisions, respectively [17–19]. Asymmetry of brain electrical activity has been reported after sports concussion, and it has been proposed that dominant asymmetry in temporal lobe activity may be a consequence of traumatic stress [20, 21] associated with dysregulation in the ANS; moreover, noninvasive measurement of asymmetry in temporal lobe high-frequency electrical activity may discriminate between sympathetic and parasympathetic tendencies in autonomic cardiovascular regulation [22]. Auto-calibration of neural oscillations may also involve optimization of ratios of energy across the brain electrical frequency spectrum, which may be important in light of evidence suggesting that individuals with PPCS have relatively greater amplitudes in lower frequency ranges (1.5 to 5 Hz) [23].

We hypothesized that the use of HIRREM by athletes with PPCS would be associated with reduction in self-reported symptom scores, improved autonomic cardiovascular regulation, and improved reaction testing.

## Methods

### Participants

The present case series is drawn from an ongoing, open-label, single-site feasibility study of HIRREM for individuals age 11 or older with diverse neurological, cardiovascular, and psychophysiological conditions (ClinicalTrials.gov Identifier: NCT02709369). The study was performed in accordance with the ethical standards of the Declaration of Helsinki and was approved by the Institutional Review Board of Wake Forest University Health Sciences. Study participation includes a baseline enrollment visit consisting of informed consent, completion of self-report inventories, short-term recording of blood pressure and heart rate, and a HIRREM assessment. Subsequently, subjects undergo a series of HIRREM sessions, followed by up to two post-HIRREM data collection visits (see below for details). The ongoing need for the use of benzodiazepine, anti-psychotic, anti-epileptic, or opioid analgesic medications is an exclusion criterion to participation. Through referrals and informal networks at a university medical center, 16 athletes with PPCS who had been involved in athletics at the club, high school, and collegiate levels were alerted to the study and screened for eligibility. One was excluded because of academic scheduling conflicts, and 15 provided informed consent (or assent, for minors) to enroll. Seven participants were females, and their mean age was 18.1 years (SD 2.6). They reported 2.7 (SD 1.8) prior concussions, symptom duration of 4.6 (SD 3.4) months since the last concussion, and at least 10 days elapsed since the last concussion. All reported prior treatment with rest, medications, vestibular therapy, or other modalities.

### HIRREM Assessment and Sessions

Each participant had a baseline HIRREM assessment (45 min) to obtain information regarding patterns of brain electrical rhythms, especially with respect to asymmetry and proportionation of energy along the frequency spectrum [14]. A series of two-channel, 3-min recordings were acquired from at least six locations on the scalp (F3/F4, C3/C4, T3/T4, P3/P4, FZ/OZ, O1/O2), with the participant at rest and while carrying out a task. Recording at each location consisted of 1 min with the eyes closed, 1 min eyes partially open, and 1 min eyes open while performing a specific mental task (e.g., recalling numbers, reading a passage). Data from the assessment were used to identify specific protocols for the initial HIRREM session.

The HIRREM sessions were scheduled to maximize frequency and efficiency, with participants generally completing two sessions in a half day, separated by a 20- to 30-min break. Each session lasted approximately 90–120 min and consisted of five to nine individualized HIRREM protocols. A protocol is a combination of sensor montage and specific software design. During protocols, brain electrical activity is recorded noninvasively and subject to high-resolution spectral analysis. A proprietary mathematical algorithm selects a dominant brain frequency for translation into an acoustic stimulus, which is delivered back to the user through earphones (Creative EP-630 or Sony Stereo Headphones MDR-EX58V) with as little as an 8-ms delay. For each session, a set of protocols is chosen by the technologist to permit “acoustic mirroring” for multiple cortical locations and frequency bands during the session. Protocols lasted from 6 to 40 min and were done with eyes either closed or open depending on the function of the cortical region or the client’s subjective status (eyes closed to facilitate relaxation; eyes open in frontal regions for executive management), all with the participant sitting or reclining comfortably in a chair.

The HIRREM process is individualized for each recipient, such that the specific protocols chosen, the session length, and the total number of sessions are variable. Technologists timed sessions and chose protocols to facilitate an overall trend toward greater hemispheric symmetry and more optimal proportionation in frequency ranges, between and within cortical regions, based upon data from the initial assessment and the ensuing sessions [14]. Participants received a mean of 18.7 (SD 6.0) HIRREM sessions, over a mean of 29.6 (SD 23.2) days, with 11.3 (SD 4.6) days of actually coming to the study site to receive the intervention. Ten participants had at least one break in sessions (defined as a period with at least 5 days between sessions), and four participants had at least two breaks.

### Rating Scales

Self-reported symptoms were recorded on the day of the HIRREM assessment and also during a post-HIRREM data collection visit that took place a mean of 19.5 (SD 23.1) days following the last HIRREM session, with sensitivity to academic scheduling constraints. The Rivermead Post-Concussion Symptoms Questionnaire (RPQ) is a 16-item survey that assesses the severity of the most common post-concussion symptoms on a scale of 0 to 4, with a total score range from 0 to 64 (least to greatest symptom severity). Items are compared to levels before the head injury and are reported as a 24-h recall [24]. The Insomnia Severity Index (ISI) is a 7-item survey that assesses the severity, nature, and impact of insomnia symptoms on quality of life over the previous 2 weeks [25]. It is scored on a five-point Likert scale from 0 (no problem) to 4 (very severe problem) on a composite score range from 0 to 28. Composite scores can be stratified into the following clinical severities of insomnia: absence (0–7), subthreshold (8–14), moderate (15–21), and severe (22–28). Its internal consistency was found to be 0.74, and a correlation with sleep diaries was also established [26]. The Center for Epidemiologic Studies Depression Scale (CES-D) is a 20-item survey that screens for risk of depression on the basis of affective depressive symptomatology [27]. Each question identifies a depressive symptom and is scored on a four-point Likert scale from 0 (“rarely”; <1 day/week) to 3 (“most or all of the time”; 5–7 days/week). Four questions have positive valence with reverse scoring. The cumulative score ranges from 0 to 60 with a score of 16 commonly used as a clinically relevant cutoff. Its internal consistency varies by demographics, with alpha coefficients between 0.60 and 0.90 and 3-month test-retest validity above 0.60 [28, 29]. Because of changes in procedures for the larger parent cohort study over the duration of the present case series, not all participants provided data for the RPQ and the CES-D.

### Assessment of Autonomic Functioning

Blood pressure (BP) and heart rate (HR) were acquired from 10-min recordings of noninvasive finger arterial pressure measurements and electrocardiography, with participants breathing at their spontaneous rate while lying quietly, supine. These recordings were obtained at the pre-HIRREM enrollment visit, just prior to the assessment of the pattern of brain electrical frequencies and amplitudes, and again during the post-HIRREM data collection visit. Systolic BP and beat-to-beat, RR intervals (RRI) files generated via the data acquisition system (BIOPAC acquisition system and Acknowledge 4.2 software, Santa Barbara, CA), at 1000 Hz, were analyzed using Nevrokard BRS software (Nevrokard BRS, Medistar, Ljubljana, Slovenia). Analysis was conducted on the first complete 5-min epoch that was considered to be acceptable for analysis. For calculation of standard deviation of beat-to-beat interval (SDNN), the RRI were visually inspected, and the data considered as artifact were manually removed. Evaluation included measures of spontaneous baroreflex sensitivity (BRS), in the frequency domain as high-frequency (HF) alpha index and in the time domain as Sequence BRS Up, Down, and All [30, 31]. Heart rate variability (HRV) was derived in the time domain as SDNN and root mean square of the difference of successive intervals (RMSSD) and in the spectral domain as absolute low- and high-frequency power.

### Reaction Testing

For seven participants, reaction testing was performed during the baseline assessment and post-HIRREM data collection visits using a drop-stick apparatus that has been validated as a way to quantify the impact of athletic concussion on psychomotor performance [32]. Following two practice trials, participants performed eight trials for measurement, and a mean distance value was calculated.

### Statistical Analysis

For mean comparisons, two-tailed paired *t* tests were performed to evaluate pre- to post-HIRREM changes. In consideration of the sample size, the nonparametric Wilcoxon signed-rank test was used to corroborate the *t* test findings.

### Physical Activity and Return to Play

Participants were asked about their physical activity level including participation in competitive athletics at two time points: their post-HIRREM data collection visit and in the course of additional follow-up data collection (phone call, email, or office visit) that took place 1 to 3 months after the post-HIRREM data collection visit.

## Results

**Fig. 1:**
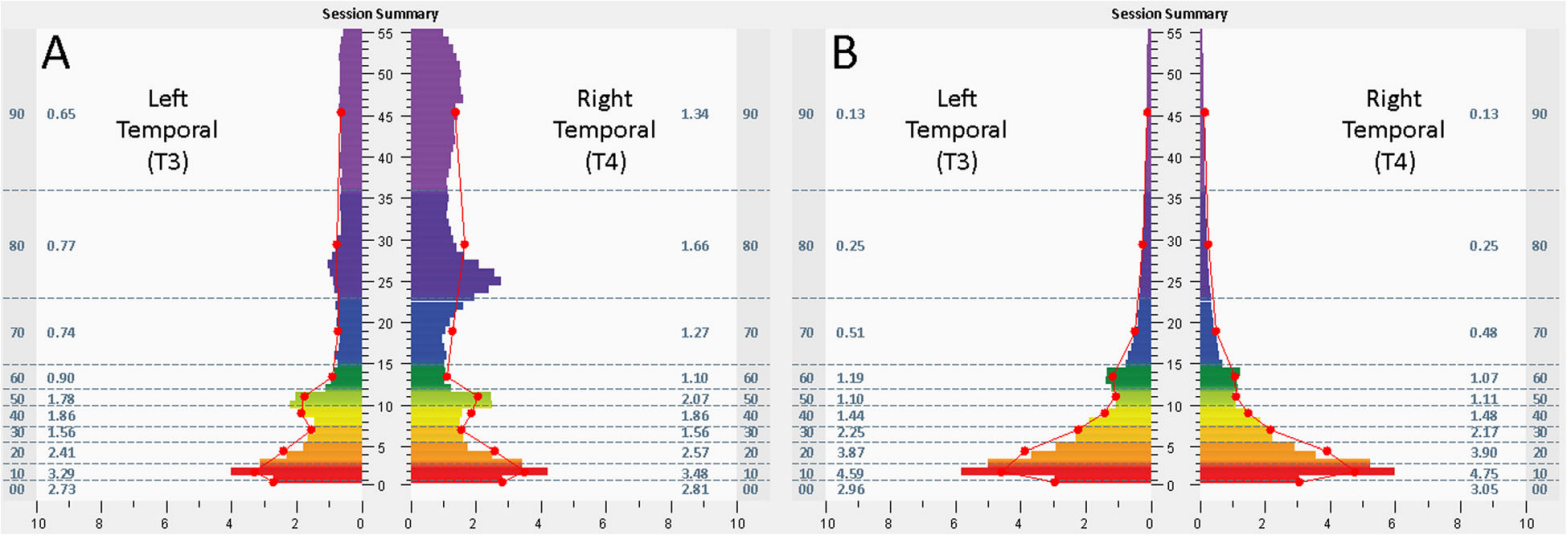
**a**, **b** Example of a FFT spectral display of brain electrical activity, this for a 16-year-old male participant, with frequency (Hz, central *Y*-axis) plotted against amplitude (μV, *X*-axis). *Color bars* represent 1-min averages of amplitudes recorded from the left and right temporal lobes (T3/T4 montage, eyes closed, *left/right boxes* of each figure, respectively) in ten frequency bins (labeled 00 through 90) from 0 through 55 Hz, at the baseline assessment (2-A) and at the penultimate minute of the 21st session (2-B). High-frequency amplitudes are more symmetrical and of reduced magnitude after HIRREM

Table 1 summarizes the study participants with respect to their demographic profile, concussion history, number of HIRREM sessions, and subsequent return to athletic activity. Table 2 shows the mean change in self-report measures from baseline to post-HIRREM assessments. Total scores for all three self-report measures, and 14 of the 16 individual items of the RPQ, showed statistically significant improvements. Table 3 shows changes in measures of BRS and HRV. All measures of autonomic cardiovascular regulation increased, with several measures showing statistically significant change (Sequence Down, Sequence Up, Sequence All, and SDNN). Mean distance on the drop-stick reaction testing improved from 23.8 (SD 5.6) cm at baseline to 19.8 (SD 4.6) cm following HIRREM (*p* = 0.044; Wilcoxon *p* = 0.016). HIRREM was well tolerated, with no adverse events reported and no dropouts. For illustrative purposes, Fig. 1 represents averaged brain electrical activity at the left and right temporal lobes at baseline (a) and during the penultimate minute of the penultimate (21st) session (b), in a single study participant, showing movement toward greater left-right symmetry in amplitudes across the frequency spectrum and also reduction of amplitudes in higher frequency ranges.

**Table 1:** Subjects’ concussion characteristics, number of HIRREM sessions, and post-HIRREM athletic participation status

Gender	Age	Number of concussions and primary athletic involvement	Months since the last concussion	Number of HIRREM sessions	Days from the final HIRREM session to post-HIRREM data collection	Post-HIRREM activity and participation status
Female	23	7; soccer	6	23	0	FE, EC
Male	20	2; baseball	9	36	16	FE, RA
Female	20	4; soccer	4	19	5	FE, RA
Male	22	2; basketball	9	16	2	FE, RA
Female	15	1; gymnastics	6	18	12	FE, RA
Female	15	1; soccer	5	26	6	FE, RA
Male	17	2; snowboarding	6	13	10	FE, RA
Male	18	3; football	11	17	27	FE, TM
Male	16	6; basketball	2	22	0	FE, RA
Female	16	3; cheerleading	0.75	16	61	FE, RA
Male	14	4; soccer	0.5	15	25	FE, RA
Female	19	1; lacrosse	6	15	15	FE, TM
Male	18	1; football	0.5	16	37	FE, EC
Male	19	2; cycling	2	14	0	FE, RA
Female	19	2; basketball	1	15	77	FE, EC

*FE* returned to full exercise, workouts, and recreational activity, *RA* returned to full participation in their athletic activity, *TM* transformed migraine with persisting mild daily headache, precluded return to competition, *EC* educational choice to not return to full participation in their athletic activity

**Table 2:** Self-report measures before and after HIRREM

Instrument (number of participants with data)	Baseline mean (SD)	Mean change after HIRREM (SD)	Paired *t* test *p* values	Wilcoxon *p* values
RPQ total (*n* = 12)	29.2 (14.9)	−19.7 (11.4)	<0.001	0.001
Headaches	3.0 (0.9)	−1.4 (1.3)	0.003	0.004
Dizziness	1.8 (1.4)	−1.1 (1.1)	0.005	0.014
Nausea/vomiting	1.3 (1.1)	−1.2 (1.0)	0.002	0.008
Noise sensitivity	1.7 (1.2)	−1.2 (0.7)	<0.001	0.002
Sleep disturbance	1.4 (1.2)	−0.8 (0.8)	0.005	0.016
Fatigue	2.3 (1.4)	−1.9 (1.1)	<0.001	0.002
Irritability/anger	1.7 (1.3)	−1.0 (1.2)	0.015	0.031
Depressed/tearful	1.4 (1.5)	−0.8 (1.5)	0.085	0.125
Frustrated/impatient	2.1 (1.4)	−1.3 (1.6)	0.016	0.022
Forgetful/poor memory	2.2 (1.6)	−1.7 (1.4)	0.002	0.008
Poor concentration	2.6 (1.4)	−1.9 (1.5)	0.001	0.008
Taking longer to think	2.4 (1.6)	−1.8 (1.3)	<0.001	0.001
Blurred vision	1.2 (1.3)	−0.8 (0.9)	0.011	0.031
Light sensitivity	1.9 (1.2)	−1.3 (0.8)	<0.001	0.002
Double vision	0.8 (1.2)	−0.5 (1.0)	0.111	0.188
Restlessness	1.4 (1.1)	−0.9 (0.9)	0.005	0.016
ISI (*n* = 15)	7.5 (4.1)	−4.1 (4.1)	0.002	0.003
CES-D (*n* = 10)	20.8 (14.2)	−12.0 (10.0)	0.004	0.004

**Table 3:** Measures of autonomic cardiovascular regulation before and after HIRREM

Measure	Before HIRREM mean ± SE	After HIRREM mean ± SE	Paired *t* test *p* values	Wilcoxon *p* values
BRS Sequence Up (ms/mmHg)	23.5 ± 3.3	32.3 ± 5.2	0.019	0.041
BRS Sequence Down (ms/mmHg)	19.9 ± 2.4	34.0 ± 6.0	0.005	0.004
BRS Sequence All (ms/mmHg)	21.9 ± 2.6	43.1 ± 10.6	0.034	0.002
HF alpha (ms/mmHg)	25.3 ± 2.6	37.4 ± 5.6	0.027	0.073
SDNN (ms)	57.1 ± 4.0	71.2 ± 7.1	0.023	0.005
RMSSD (ms)	54.9 ± 6.0	61.6 ± 7.7	>0.2	>0.2
LF (ms^2^)	1397 ± 223	1840 ± 368	0.131	0.188
HF (ms^2^)	1715 ± 465	2155 ± 564	>0.2	>0.2

Following use of HIRREM, all 15 athletes returned to full exercise, workouts, and recreational activities, as well as return to learning and academic activities, and ten athletes returned to full participation in their respective sport. In accordance with current concussion management consensus practices [1], two college athletes were not allowed to return to full participation in their respective sport due to persisting, mild, chronic daily headaches. By history, both appeared to have had a pre-existing vascular headache condition and thus to possibly have experienced transformed migraine associated with the concussion. Three athletes resumed full exercise and workouts but chose to pursue educational opportunities rather than return to active participation in their team sport.

## Discussion

In this case series of young athletes with PPCS, the use of HIRREM was associated with significant reductions in concussion-related symptomatology including both physical and emotional symptoms, improvements in measures of autonomic cardiovascular regulation and reaction time, and for the majority, return to participation in competitive athletics. We are not aware of a precedent for demonstration of this full constellation of outcomes within a relatively short time frame after use of an intervention for individuals with PPCS. Nonetheless, the open-label nature of the study and the absence of a control group preclude definitive inference that the outcomes were a direct consequence of the HIRREM intervention.

A major question is whether, or to what degree, the outcomes may be attributed to placebo. The “placebo effect” may comprise contributions from the natural history of the disease, reporting biases, the influence of co-interventions, and the neurobiological placebo *response* [33]. The placebo response in turn includes changes mediated by doctor-patient interaction or subjective expectation. The time course for reduction of PPCS is variable, and a symptom trajectory for participants, had they not enrolled in the study, cannot be predicted retrospectively with confidence. There is little doubt that many with PPCS are living with the burden of a long clinical course, with symptoms lasting more than 2 years in some cases [34]. Most participants in the current study had stable patterns of symptoms, persisting for many months, in spite of the use of other therapeutic strategies, yet reported not only symptom reduction but also return to full participation in their athletic activity. Return to play is an objective behavior, and thus, subjective reporting bias (for example, “social desirability response bias”) would not appear to have been a major contributor to this outcome. Further, efficacious treatments for PPCS are largely wanting, and the 2012 Zurich guidelines specifically state that medications should not be used to mask symptoms that would otherwise prevent return to play. Thus, it would not seem likely that these results reflect primarily the natural history of the disease or that a co-intervention was the cause for these athletes’ return to play.

Although changes in the self-report questionnaires may be subject to reporting biases, the mean change in the RPQ (−19.7, SD 11.4) was notably larger than the change in the RPQ reported for both the active (−5.4) and sham (−7.0) intervention groups, from a recently published clinical trial for PPCS [35]. Components of the placebo *response*—especially doctor-patient relationships and subjective expectations—can facilitate robust and wide-ranging improvements in health. However, placebo responses in clinical studies are reportedly more prominent for continuous and subjective outcomes, compared to binary or objective outcomes [36]. Since return to competitive athletic activity can be considered a binary and objective outcome, with changes in HRV and reaction testing also being measured objectively, these findings do not appear to be consistent with typical characteristics of the placebo response. All told, it appears unlikely that the placebo phenomenon was the principal cause for return to play in these athletes.

There are other reports of promising results from interventions involving auto-regulatory strategies as a means to treat PPCS. For example, athletes with PPCS who participated in a graded aerobic exercise training program (five to six sessions per week until they were capable of exercise to voluntary exhaustion without symptoms) demonstrated significant reductions in symptoms and increased peak heart rate at maximal exercise [37]. Four to 16 weeks of subsystem aerobic exacerbation was recently reported to be superior to a full-body stretching program for reducing PPCS in adolescents [38]. A comprehensive program including graded aerobic exercise and visualization has been found to facilitate improvements for children with PPCS [39]. The basis for a concussion management strategy that places emphasis on evaluation of exercise tolerance and use of exercise as a treatment option [40] is further justified by the insight that aerobic exercise may have specific beneficial effects on cerebrovascular regulation for individuals with mild traumatic brain injury [41]. Physiologically, the allostatic approach of the HIRREM intervention overlaps with the rationale for exercise treatment for PPCS, in that both embrace the importance of recovering neural and cardiovascular regulatory competence.

## Conclusions

In conclusion, this exploratory study found that the use of an allostatic, noninvasive, closed-loop, acoustic stimulation neurotechnology, HIRREM, by a series of young athletes with PPCS, was associated with significant reductions in clinical symptoms; improvements in autonomic cardiovascular regulation; return to exercise, recreational, and academic activities; and a high rate of return to play. The findings do not appear to be consistent with the natural history of the disease, doctor-patient interaction, subjective expectation, or other placebo components. A larger controlled study is warranted.

## Abbreviations

ANS: Autonomic nervous system
BP: Blood pressure
BRS: Baroreflex sensitivity
CES-D: Center for Epidemiologic Studies Depression Scale
HF: High frequency
HIRREM: High-resolution, relational, resonance-based, electroencephalic mirroring
HRV: Heart rate variability
ISI: Insomnia Severity Index
mTBI: Mild traumatic brain injury
PPCS: Persisting post-concussion symptoms
RPQ: Rivermead Post-Concussion Symptoms Questionnaire
RR: R wave to R wave on electrocardiogram
RRI: RR intervals
SDNN: Standard deviation of beat-to-beat interval
